# Validation of the Arabic Version of Strengths and Difficulties Questionnaire in Early Childhood Education in Qatar

**DOI:** 10.3390/children10010146

**Published:** 2023-01-11

**Authors:** Maha Al-Hendawi

**Affiliations:** Department of Psychological Science, College of Education, Qatar University, Doha P.O. Box 2713, Qatar; maha.alhendawi@qu.edu.qa; Tel.: +00974-4403-4043

**Keywords:** emotional and behavioral disorders, early childhood, SDQ, psychometrics, Qatar

## Abstract

This study examined the validity of the Arabic version of the Strengths and Difficulties Questionnaire (SQD, teacher version) among a sample of young children in Qatar. Teachers rated 502 children aged four to five years from public preschools using the SDQ teacher version. The factor structure of the SDQ was analyzed using exploratory and confirmatory factor analyses. I calculated Cronbach’s alpha coefficient and item–total correlations to determine the reliability of the five subscales and overall SDQ. The findings showed acceptable reliability, with the exception of the Peer Problems Scale. Common fit statistics—including the comparative fit index, non-normed fit index, and goodness-of-fit index—were used for the confirmatory factor analysis. In general, satisfactory psychometric characteristics were observed for the preschool SDQ, suggesting that the questionnaire could be administered to preschool-age children in Qatar.

## 1. Introduction

Psychosocial disorders in children are widespread, with a prevalence of up to 34.7% [1]. These disorders have both long- and short-term repercussions, and early recognition may have a significant and beneficial effect on their management [2,3]. However, investigating psychological disorders, especially in young infants, presents various execution and reliability challenges. The availability of a well-validated instrument that is simple to use with young children helps mitigate these problems to a significant degree.

The Strengths and Difficulties Questionnaire (SDQ) is a prominent instrument for evaluating mental health and behavioral issues in children and adolescents. It was designed by Goodman (1997) in the United Kingdom [4] and is currently used in both research and therapeutic settings for children aged 2–17 [5]. Compared to other forms of screening, such as the Child Behavior Checklist [6], the SDQ has several benefits, such as brevity and practical assessment of attention deficit and hyperactivity; thus, it has been translated into other languages and used worldwide, mainly because of its focus on both competencies and difficulties and its brevity (SDQ.; Goodman, 1997; see https://www.sdqinfo.com/, accessed on 7 February 2022).

Studies indicate that the predictive and concurrent validity of the SDQ is generally good [7,8]. Normative SDQ data in school-age children are available in several countries [9,10,11,12,13,14]. Several studies have determined that the internal consistency of individual samples was adequate for most subscales and supported the feasibility of the screening instrument in general. Mixed findings have been reported. For instance, Tobia et al., reported that internal consistency was lower for Peer Problems and Emotional Symptoms Scales [14]. Huskey et al., discovered a similar lack of consistency in the Peer Problems Scale in seven European countries [12].

The five-factor structure of the SDQ is receiving increasing support as more studies have used confirmatory factor analysis to test its hypothesized factor structure [15]. In the U.S.; He et al. (2013) reported that the five-factor structure of the SDQ showed a satisfactory fit to the data and was invariant across age, ethnicity, gender, and income subgroups [11]. The SDQ scores showed a noticeably higher likelihood of fitting the DSM-IV criteria for diagnosis. In Asia, the CFA of the SDQ supports a five-factor structure [13]. The psychometric features of the SDQ for preschoolers (ages 3–5) have been the subject of a very small number of investigations, and these studies were mainly conducted in the West [3,16,17]. Their findings demonstrated that the SDQ is suitable as a psychopathological screening tool in linguistically and culturally varied preschool settings and that the SDQ fits the five-factor model in all preschool groups. One study conducted in South Africa on young children aged 4–6 years [18] reported mixed findings that some subscales did not work in their context and language; in particular, Conduct Problems Scale was only partially supported, and both the Hyperactivity/Inattentive and Peer Problems subscales were poorly loaded [18].

Although a great deal of research has been undertaken on the reliability and validity of the SDQ, several critical areas still require additional exploration [19]. First, while reliability has been widely investigated, the dependability of the subscales, particularly the Conduct Problem Scale issues and Peer Problems Scale, seems inadequate. Additionally, the reliability of the test-retest and the temporal stability were found to be adequate and not high in a recent systematic review; on the other hand, the sensitivity and the positive predictive value were unsatisfactory [15]. Some studies in Arab countries have used the SDQ [20,21,22]; however, there has been little work on the psychometric characteristics of the instrument for use in Gulf nations, focusing mainly on young children.

Two to four percent of preschoolers display clinically significant levels of disruptive behavior that warrant a diagnosis of attention deficit hyperactivity disorder (ADHD) or conduct disorder, and the expulsion rate for preschoolers is three times higher than that of students in higher grades [23,24,25]. The validation of SDQ in preschoolers can be of great help [7]. It should be noted that although emotional and behavioral difficulties are quite common among preschoolers, particularly in economically disadvantaged communities, very few really get any help due to delays in diagnosis.

## 2. Literature Review

There is common agreement on the importance of early diagnosis and early interventions for behavioral and emotional problems [3]. Approximately 10–30% of preschool children exhibit high levels of disruptive behavior [26]. Preschool children are expelled approximately three times more often than children in grades K–12; approximately 2–4% of preschoolers are reported to exhibit disruptive behaviors to a clinical degree and are diagnosed with attention deficit hyperactivity disorder (ADHD) or oppositional defiance disorder [23,24]. Yoder and Williford (2019) reported that teachers rated approximately one-fourth of their study sample (*N* = 2427 preschoolers) as exhibiting high levels of problem behavior; 16% of the total sample were described as showing these behaviors at a clinical level (i.e., indicated by DSM-5 symptom-count criteria for ODD and ADHD) [25]. Therefore, screening instruments, especially at this early stage, play a significant role in measuring the different types of behavioral and psychosocial problems, the strengths that might be found, and the severity of these problems [7].

### 2.1. Related Studies

Many studies have examined the psychometric properties of different versions of the SDQ in children and young people. These studies generally support the feasibility of this screening instrument. For example, Husky et al. (2018) examined and compared the internal consistency of teacher and parent versions in their study of 541 children aged 5–12 years in seven European countries [12]. They found that the internal consistency of the total sample was adequate for most subscales, except for Peer Problems. The study by Tobia et al. (2018), in which 301 teachers assessed 3302 children aged 3 to 15 years, validated the internal structure of the Italian teacher version of the SDQ [14]. Internal consistency was lower for the Emotional Symptoms and Peer Problems Scales. Thus, the psychometric properties of the teacher version for this Italian sample were only partially consistent with those of previous studies in other countries. Español-Martín and colleagues (2021) assessed the reliability and validity of the Spanish versions of the SDQ (teacher, parent, self-report) with a sample of 6775 students ages 5 to 17 [10]. The results demonstrated acceptable reliability estimates for all SDQ subscales, and the CFA supported the original five-factor model. In the US, He et al. (2013) evaluated the five-factor structure of the SDQ and assessed its convergent validity compared to comprehensive clinical diagnostic assessments using data from the National Comorbidity Survey-Adolescent Supplement, a nationally representative sample of adolescents between 13 and 18 years of age (*N* = 6483) [11].

The findings indicated that the five-factor structure of the SDQ showed a satisfactory fit to the data and was invariant between the age, ethnicity, sex, and income subgroups. The SDQ scores indicated a significantly increased probability of meeting the DSM-IV criteria for a disorder. Numerous studies in Asia have evaluated the validity and reliability of the SDQ. Shibata et al. (2015) used the parent and teacher versions of the SDQ in Japan with a sample of 1487 elementary school children aged 6 to 12 years [13]. The CFA results of these SDQ reports support a five-factor structure. Du et al. (2008) described the normative data, validity, and reliability of three Chinese versions of the SDQ in a large sample of children in Shanghai of 2128 students between the ages of 3 and 17 [9], providing only partial support for structural validity but good support for convergent validity.

### 2.2. Arabic Version of the Instrument

Arabic versions of SDQ have demonstrated good psychometric properties [27]. Although several studies in Arab countries have used SDQ [20,21,22,28,29], research on the psychometric properties of the instrument for Gulf countries, especially for young children, is limited. Only Ababneh and Alomari (2016) investigated the psychometric properties of the teacher version of the SDQ for children aged four and five years in Jordan (*N* = 788) [28]. They conducted a factor analysis of three factors that correspond to the three dimensions of the psychometric properties of the instrument. They also evaluated concurrent validity with an early development instrument (Cronbach’s α = 0.73). Other studies have focused on older populations, and some studies have focused on determining the prevalence of emotional and behavioral disorders. Maajeeny (2019) aimed to extend previous efforts to determine the prevalence of behavioral and emotional disorders among children in Saudi Arabia to evaluate the demand for intervention services [22]. He distributed the SDQ to teachers of students aged 4–17 years and concluded that students with emotional and behavioral disorders in Saudi Arabia might represent around 25% of this age group. The results of the SDQ reliability test showed an acceptable level of general reliability (Cronbach’s α = 0.64) [22]. El-Keshky and Emam (2015) conducted a cross-cultural examination of the teacher version of the SDQ in Saudi Arabia (*n* = 323) and Oman (*n* = 439), focusing on emotional and behavioral difficulties in children diagnosed with learning disabilities [20]. Researchers have also examined the structure of the SDQ. Multigroup CFAs based on structural equation modeling indicated cultural invariance for the instrument. The three-factor model was supported and provided a better explanation of the structure of the SDQ. El-Keshky and Emam found that the Arabic version of the SDQ has acceptable psychometric properties and performs well and consistently across different Arab cultures and genders. Al-Mukhani et al. had 377 students aged 11 to 16 who completed the SDQ [29]. They concluded that the self-reporting version of the SDQ is a reliable screening instrument for behavioral and psychological problems in children in Oman. Emam et al. (2016) targeted middle school students (*N* = 815). Their assessment of the five-factor SDQ structure revealed significant empirical support. Their findings revealed a reasonable fit between the three forms of informants. Across genders, factor variances, factor correlations, and item residuals were not invariant.

### 2.3. Early Childhood Studies

Research on the psychometric properties of SDQ for preschool children (ages 3–5) is limited [3,17]. In a Dutch community sample focusing on children 4 to 7 years of age, Stone et al. (2015) examined the SDQ versions of the parent (*n* = 1513) and the teacher (*n* = 2238) [7]. They tested reliability and construct validity and examined predictive validity. They found that the five-factor structure was supported for both parent and teacher versions, and the indices of the validity of the criteria were adequate. Dahlberg and colleagues (2019) examined the SDQ of 3 to 5 years old in a sample of (*N* = 17,752) in Sweden [3]. Their results revealed that the original five-factor SDQ model could be used for younger populations. It also demonstrated good construct validity of the SDQ for a preschool population and suggested that the instrument can be used to evaluate children’s problem behavior from various informant perspectives. Downs et al. (2012) examined SDQ for (*N* = 477) children from the United States (*n* = 298; ages 3 to 5 years) and Germany (*n* = 179; ages 3–6 years) [30]. The SDQ showed adequate validity and reliability in different cultures and linguistics. The results generally support the suitability of SDQ as a psychopathological screening tool in culturally and linguistically diverse preschool settings. The results supported the fit of the SDQ five-factor model in all three groups of preschoolers. Croft et al. (2015) tested the SDQ for ages three to four years [16]. They were completed by 16,659 parents collected as part of the Millennium Cohort Study, which followed the development of children born in the UK during 2000–2001. The CFA indicated the adequacy of the five-factor measurement model. They concluded that the satisfactory psychometric properties of the preschool version supported its use as a screening tool to detect behavioral and emotional problems in children aged 3 to 5 years. Mellins et al. (2018) also examined the psychometric properties of SDQ, using data extracted from a large population (*N* = 1581) of young children aged 4 to 6 years in South Africa [18]. Although the total difficulty score, Emotional Symptoms Scale, and Prosocial behavior factors were confirmed, behavior problems were only partially supported. (The Hyperactivity/Inattentive and Peer Problems subscales loaded poorly). The authors concluded that, although the SDQ might be a valuable screening instrument in South Africa, some subscales did not work in this context and language; additional measures or modifications could be needed.

### 2.4. The Current Study

The current research study aimed to assess the teacher version of the SDQ’s reliability, inter-measure correlations (Cronbach’s alpha), discriminate validity, as well as conceptual, exploratory (EFA), and confirmatory factor analysis (CFA) for pre-kindergarten and kindergarten children in Qatar. There have not been many recent studies on the psychometric properties of the SDQ teacher version in early childhood populations, especially in the Arab world. The study of the psychometric characteristics of the SDQ teacher version and the validity of its usage in children of this age group might pave the way for the validation of the use of this instrument broadly by teachers in Qatar’s public schools. This, in turn, can aid in the early diagnosis of emotional and behavioral disorders in children. This research study aimed to answer the following questions. One is to determine whether the Arabic-version of SDQ (teacher version) is a reliable instrument to assess preschoolers in Qatar, and the second is to determine whether data from this study can be used to apply the five-factor SDQ framework. For this purpose, I conducted a validation and psychometric assessment of the SDQ teacher report version in a group of four to five-year-old children. To my knowledge, this is the first study from Qatar to analyze the factor structure of SDQ (teacher version) in a representative sample of preschoolers.

## 3. Methodology

### 3.1. Participants

In collaboration with the Ministry of Education and Higher Education, we recruited participants from six preschools in different municipalities. The total number of children in these six preschools was 591 (292 boys and 299 girls). All preschools’ teachers were invited to information sessions. Twenty-five teachers completed the SDQ and the demographic information forms for 502 children aged four or five years (including boys, 51.4%, and girls, 48.6%). The project was approved by the ethical review board at Qatar University as well as the Ministry of Education and Higher Education in Qatar.

The demographic variables of the participating children were divided according to sex, age, and grade, and the frequency and percentage of each variable were calculated (see Table 1).

### 3.2. Measures

The SDQ assesses the psychological adjustment of children and adolescents aged 2–17 years and can be used for screening in clinical evaluations and research [4]. It has 25 questions evenly divided among five categories that evaluate emotional symptoms, behavior difficulties, lack of attention or hyperactivity, peer problems, and prosocial behaviors. The overall difficulty score is generated by subtracting the Prosocial Scale from the subscales and reflects the degree and type of psychological and social challenges [7]. The SDQ is a short questionnaire that consists of the same 25 questions but may be answered in one of three distinct ways: by a parent, by a teacher, or by the child himself, if they are older. All SDQ variants used the same items.

For this study, I used the SDQ for Arabic teachers to evaluate the psychological and behavioral problems of young children. The SDQ consists of 25 questions scored on a Likert scale, with zero representing not true, one representing somewhat true, and two representing absolutely true. Most of the items in the SDQ are negatively worded; only five items are positively worded, so they reverse the score.

In the SDQ, established by Goodman (1997), there are five scales, and each scale has five components that constitute the scale. The overall score on each of the five subscales may range from 0 to 10 points while the total score on the total difficulty scale, which comprises 20 elements, can range from 0 to 40 points. When it comes to the four problem scales, higher scores imply more difficulties, but when it comes to the Prosocial and Conduct Problems Scale, higher values indicate a stronger score on Prosocial Scale.

### 3.3. Theoretical Background

The EFA and the CFA were used to examine the validity of the psychometric properties of the teacher version of the SDQ and its use in preschoolers. EFA accounts for the correlations between observable variables, focusing on manifest variables that can be reduced to fewer hidden variables, termed constructs [31]. The EFA can be used to evaluate the validity and reliability of the scale used to measure the outcome of interest [32]. Kaiser-Meyer-Olkin (KMO) and Bartlett’s sphericity tests are essential to determine the validity and robustness of the EFA. Factor loading is the correlation between a specific observable variable and a particular factor, with higher values indicating stronger relationships. After the EFA determines the factor structure, the CFA model is used to confirm the factor loading pattern and the number of underlying factors.

### 3.4. Data Analysis

I determined the means of each of the 25 items on the scale and the standard deviations for those means. G*Power software was used to calculate the sample size and power [33]. In addition, I evaluated the SDQ’s internal reliability with the Qatari population using Cronbach’s alpha to analyze each of the five scales and the Total Difficulties scale separately. To assess the factorial structure of SDQ in this population, I conducted an EFA. Two tests, Bartlett’s test of sphericity and the Kaiser-Meyer-Olkin measure of sampling adequacy, were used to determine if these data were suitable for component analysis. To support the EFA results and assess the factorial structure of the SDQ, I conducted CFA on this dataset using EQS 6.1. software. The goodness-of-fit, non-normed, and comparative fit indices were used as standard fit statistics.

## 4. Results and Discussions

Findings of this study represent a statistical analysis of the administration of SDQ to 502 teachers in Qatar. First, the data were checked and cleaned for possible errors, extreme values, and outliers. The minimum sample size required for this effect size of 0.3 was N = 63 (α = 0.05 and power = 0.80). Thus, a sample size of 502 was sufficient to test the hypothesis of the study.

### 4.1. Descriptive Statistics

The means and standard deviations for each of the 25 items on the scale are shown in Table 2. The mean values for each item were different, ranging from very low values, close to zero (items 3, 5, 12, 16, 19, and 22), to high values, above 1 (e.g., Items 1, 4, 9, 17, 20).

### 4.2. Discriminate Validity

The relationship between each item and the total scale score showed discriminant validity. Most items on the scale were positively and significantly correlated with the total score (*p* < 0.01). This means that these elements improved the validity of the scale in the current data. However, some items (i.e., 1, 4, 9, 17, and 20) correlated with the total score. This indicates that these items did not work in the same direction as the others and negatively affected the discriminant validity of the scale.

### 4.3. Reliability and Inter-Measures Correlations

The reliability values of all scales were good, in addition to the Peer Problems Scale (Cronbach’s α = 0.29; see Table 3). This low reliability is likely to affect the factorial structure of this scale. I also calculated the correlations between the measures on the scale and all correlation values were statistically significant (*p* < 0.01). As expected, the correlation was positive for the four difficulty Scales and negative for the Prosocial Scale.

### 4.4. Exploratory Factor Analysis

Several studies have assessed the factorial structure of the SDQ [14,34]. The Kaiser-Meyer-Olkin sample adequacy score was 0.89, and Bartlett’s test of sphericity was statistically significant (*p* < 0.001). These findings indicate that the present data are adequate for factor analysis. Using an eigenvalue greater than 1.00, the EFA results indicated that five factors were extracted from the data. The eigenvalues and percentages of the variance explained by each extracted factor are presented in Table 4. These five factors explained approximately 60% of the variance.

The five extracted factors/scales and item loadings are listed in Table 5. The first scale (that is, the emotional Symptoms Scale) was clearly extracted, and its original five items were highly loaded (loadings > 0.30). However, two items (somatic symptoms and nervousness in new situations) were loaded on the Peer Problems Scale. The Conduct Problems Scale was also extracted with its five original items with high loadings, except for those relating to tempers. As mentioned above, positively phrased items operate differently from negatively phrased ones. Indeed, all positively worded items on the scale loaded highly on the Prosocial Scale. I also extracted a Hyperactivity/Inattention Scale from the five original items. All of these items had high loadings, but the two positively worded items were also highly loaded on the Prosocial Scale. The fourth scale (Peer Problems) was not extracted, whereas the Prosocial Scale was extracted with its five original items and high loadings (all loadings ≥ 0.70).

### 4.5. Confirmatory Factor Analysis

I performed CFA on this dataset to support the EFA findings and to analyze the factor validity of the SDQ. Index values greater than or equal to 0.90 indicate a satisfactory level of fit for these three indices. In addition, I used the standardized root mean square residual and root mean square error in the approximation. Values of these indices that were equal to or lower than 0.05 were deemed satisfactory [35]. The findings of these indices are shown in Table 6. As can be seen, none of the goodness-of-fit ranges or values were reached by any of the fit indices, indicating that these data do not support the five-factor SDQ model.

## 5. Discussion

This study examined the validity and application of the hypothesized factor structure of the SDQ (teacher version) in a sample of preschoolers in Qatar. The findings indicate the poor internal reliability of the Peer Problems Scale. The results also suggest that, unlike several other studies, the five-factor structure does not fit well with these data. In particular, the EFA identified five factors that accounted for 60% of the variance; however, two items, namely, somatic symptoms and nervousness in new situations, had higher loadings on the Peer Problems Scale than on the Emotional Symptoms Scale. The Conduct Problems Scale had a high loading on the three items. I also extracted a Hyperactivity/Inattention Scale from the five original items. These items had high loadings, but the two positively worded items were also highly loaded on the Prosocial Scale.

In the CFA, none of the goodness-of-fit ranges or values was reached for any of the fit indices. Based on these results, I can conclude that these data do not show the original five factors of the SDQ in a straightforward manner [36,37]. Although five factors were identified in these data (with eigenvalues > 1) and four were extracted mainly from their original elements, one factor did not appear in the results. Moreover, the five positively worded items functioned in different directions than the other items on the scale. This finding suggests an effect bias. This bias is the variance due to a technique of measurement, such as item wording, as in this example favorably phrased items, instead of the conceptual framework, which is the variance due to a technique of measurement, such as item wording, as in this example favorably phrased items, instead of the conceptual framework [38]. It is noteworthy that in grades 2–4, correlations between latent components were substantial, especially when the conduct Problems Scale issue factor was included, showing a large overlap between problem areas and a modified version of Goodman’s five-factor model, which demonstrated a satisfactory fit [39].

In their meta-analysis, Stome et al., found that the bulk of the research supporting the five-factor structure was conducted on adolescents and the findings favored the parental assessment of their children’s social development using the SDQ [8]. However, in a subsequent study, Stone et al., verified the applicability of five-factor models in parent and teacher versions for children aged 4–7 [7]. Interestingly, in a study of young and old children, factor loadings were found to be acceptable for all groups, although older children judged by teachers had better internal consistency than younger children scored by their parents [40]. The authors also suggested using a customized approach when using the SDQ for low-risk epidemiological samples. In particular, the five-factor structure was not validated in a large community sample of 7–17 years old, none of the subscales being unidimensional [41]. Some other studies found little evidence for the five-component structure [42,43], revealing problems in the SDQ component structure, especially with reverse-coded questions [41], internal consistency [44], and high correlations between scales [45].

Although results of this study do not agree with those of other studies that support a five-factor model for CFA use [10,46,47], it is important to note that, in several studies, modified five-factor structure models were used and/or multiple models were found suitable. In this study, since the objective was to validate the original SDQ, I did not use any modification of the model. For instance, Boe et al., used a modified model, and Yao et al., concluded that both five-factor and higher-order structures are suitable. Boe et al., suggested that their findings would have been more positive if they had considered local dependencies within the variables [46]. The fitting of the model can be further enhanced by defining cross-loadings of favorably worded problem items on the Prosocial Scale and minor variables on the Hyperactivity Scale, as shown in a recent study in Swedish adolescents [36].

In close agreement with the findings of this study, internal consistency and the various SDQ scales were found to be good, with the exception of the peer issues scores in young children in the study conducted by Husky et al., which covered seven European countries and Hagquist et al., conducted in Sweden [12,48]. Supporting the use of specific subscales, Gustafsson et al. determined that for children ages 1–3, the subscales ‘Hyperactivity’ was valid, whereas, for ages 4–5, the full original SDQ scale, 4-factor solution, demonstrated acceptable validity [49]. Other studies have indicated that there are no accessible norm scores for the Conduct Problems Scale and Peer Problems Scale between the ages of 2–3 and 4–5 owing to low internal consistency [16,50,51].

As stated above, although adding an additional method component might enhance model fitting, I did not study techniques to overcome incorrect solutions in these models because I utilized a confirmatory approach, and the objective was to validate the original five-factor structure. I want to highlight that while the findings do not fully support the five-factor model or internal consistency for all subscales, it is crucial to note that EFA and CFA are just one type of verification approach. The use of specific scales and combinations of scales could be effective depending on the research problem. These aspects should be explored further in the future. As Karlsson et al., correctly contended, the SDQ has sufficient factorial validity for epidemiological studies if investigators realize its flaws and explain the implications of the method and related compounds [36].

### Strengths and Limitations

The key strength of this study is that it is the first study in Qatar to focus on preschoolers. The findings of this study will motivate other researchers to further explore different versions of the SDQ in different populations, which will accelerate the clinical and practical applications of this tool. This study had some limitations. First, I only included public preschools; therefore, results may not apply to private institutions. Moreover, CFA is a rigorous analysis; hence, its use may limit generalization. Self-reported data may or may not be accurate. It is also possible that the five-factor model did not fit the data because some key refinement was lost in the translation from the original SDQ to Arabic. In the future, the validation of SDQ compared to other mental health instruments will strengthen its construct validity.

## 6. Conclusions

The purpose of this research was to investigate the psychometric characteristics of the SDQ teacher version for children aged four to five years. The participants came from a variety of kindergartens in Qatar, each located in a different region. The findings provide credence for an updated version of the three-factor model, which includes an allowance for a constructively positive element. The Peer Problems subscale should be approached with care, even though I discovered a strong internal consistency. This subscale had lower internal consistency values than the others, and one of its items had a nonsignificant factor loading. This study is the first of its kind in the Gulf area to demonstrate the psychometric features of the SDQ teacher questionnaire for behavioral difficulties or emotional and behavioral disorders in preschool-aged children. The findings of this study will increase awareness that the Arabic version of SDQ can be used as a screening tool to identify child problem behaviors at an early age, which greatly helps in the development of interventions.

The findings of this study are also of high academic importance as they highlight the importance of individual subscales and their clinical implications. Future research should examine the validity of other versions of the SDQ to have multiple informants for rated behavior problems. The clinical validity of the SDQ and each of its subscales should be investigated in the future to ascertain the extent to which the instrument may be used for clinical referrals. Future research should also examine the clinical validity of the SDQ and all its subscales to determine the instrument’s potential for clinical referrals. This research study will be crucial to revising the theoretical underpinnings of the SDQ and understanding the many questions that need to be changed for various age groups and versions. Another subject that must be thoroughly researched for each item is translation since even slight changes in meaning after translation may significantly impact the response.

## Figures and Tables

**Table 1:** Demographic variables of participating children: sex, age, and grade.

Variable		Frequency	Percentage
Gender	Males	258	51.4
	Females	244	48.6
Age	4 years	227	45.2
	5 years	275	54.8
Grade	Prekindergarten	224	44.6
	Kindergarten	278	55.4

**Table 2:** Item–Total Score Correlations and Descriptive Statistics.

Item	*M*	*SD*	Item–Total Correlation
1. Considerate	1.23	0.62	−0.140
2. Restless	0.54	0.69	0.62
3. Somatic symptoms	0.12	0.38	0.49 **
4. Shares readily	1.22	0.64	−0.12 **
5. Tempers	0.14	0.40	0.52 **
6. Solitary	0.30	0.56	0.37 **
7. Obedient	0.48	0.59	0.38 **
8. Worries	0.37	0.58	0.57 **
9. Helpful if someone hurt	1.11	0.64	−0.13 **
10. Fidgety	0.52	0.68	0.66 **
11. Has good friend	0.74	0.71	0.23 **
12. Fights or bullies	0.19	0.47	0.48 **
13. Unhappy	0.29	0.56	0.52 **
14. Generally liked	0.61	0.59	0.31 **
15. Easily distracted	0.60	0.70	0.65 **
16. Nervous in new situations	0.21	0.48	0.59 **
17. Kind to younger children	1.37	0.59	−0.11 *
18. Lies or cheats	0.06	0.29	0.37 **
19. Picked on or bullied	0.03	0.20	0.35 **
20. Often volunteers	1.11	0.66	−0.13 **
21. Thinks before acting	0.82	0.62	0.42 **
22. Steals	0.07	0.30	0.37 **
23. Better with adults than with children	0.79	0.70	0.12 **
24. Many fears	0.30	0.55	0.48 **
25. Good attention	0.73	0.68	0.42 **

* *p* < 0.05. ** *p* < 0.01.

**Table 3:** Internal Reliability.

Scale	Cronbach’s Alpha	1	2	3	4	5
Emotional Symptoms	0.81	1				
Conduct Problems	0.63	0.35 **	1			
Hyperactivity-Inattention	0.81	0.38 **	0.56 **	1		
Peer Problems	0.29	0.44 **	0.30 **	0.28 **	1	
Prosocial	0.86	−0.36 **	−0.39 **	−0.48 **	−0.53 **	1
Total Difficulties	0.83	0.74 **	0.71 **	0.82 **	0.63 **	−0.59 **

** *p* < 0.01.

**Table 4:** Eigenvalue and percentage of variance explained by the extracted factors.

Component	Eigenvalue	Variance (%)	Cumulative Variance
1	7.28	29.10	29.10
2	2.76	11.05	40.16
3	2.25	9.01	49.16
4	1.36	5.43	54.59
5	1.10	4.40	58.98

**Table 5:** Extracted Factors and Loadings.

	Factor				
Item	Prosocial	Hyperactivity	Emotional	Conduct	Peer
Somatic symptoms			−0.32		−0.56
Worries			−0.76		
Unhappy			−0.70		
Nervous in new situations			−0.35		−0.43
Many fears			−0.84		
Tempers				0.11	−0.74
Obedient ^a^	0.54			0.11	
Fights or bullies				0.52	
Lies or cheats				0.72	
Steals				0.73	
Restless		0.80			
Fidgety		0.79			
Easily distracted		0.73			
Thinks before acting ^a^	0.51	0.51			
Good attention ^a^	0.48	0.50			
Solitary			−0.59		
Has good friend ^a^	0.57				
Generally liked ^a^	0.77				
Picked on or bullied				0.64	
Better with adults than with children	−0.48				
Considerate	−0.70				
Shares readily	−0.70				
Helpful if someone hurt	−0.76				
Kind to younger children	−0.76				
Often volunteers	−0.76				

^a^ Positively worded items.

**Table 6:** Goodness-of-Fit Indices of Confirmatory Factor Analysis.

Fit Index	NNFI	CFI	GFI	SRMR	RMSEA
Value	0.75	0.78	0.80	0.10	0.09
Range	≥0.90	≥0.90	≥0.90	≥0.05	≥0.05

*Note*. NNFI = non-normed fit index; CFI = comparative fit index; GFI = goodness-of-fit index; SRMR = standardized root mean square residual; RMSEA = root mean square error of approximation.

## Data Availability

Data are not publicly available due to ethical restrictions.
